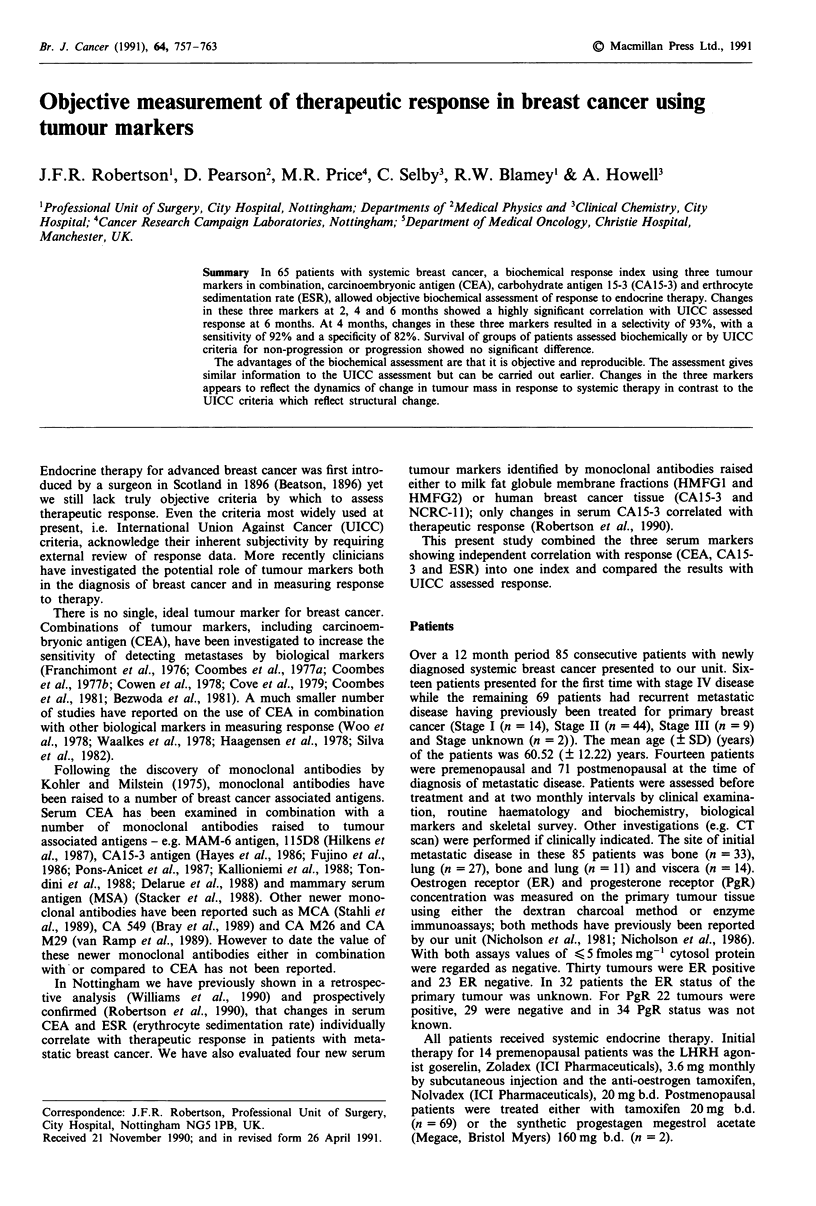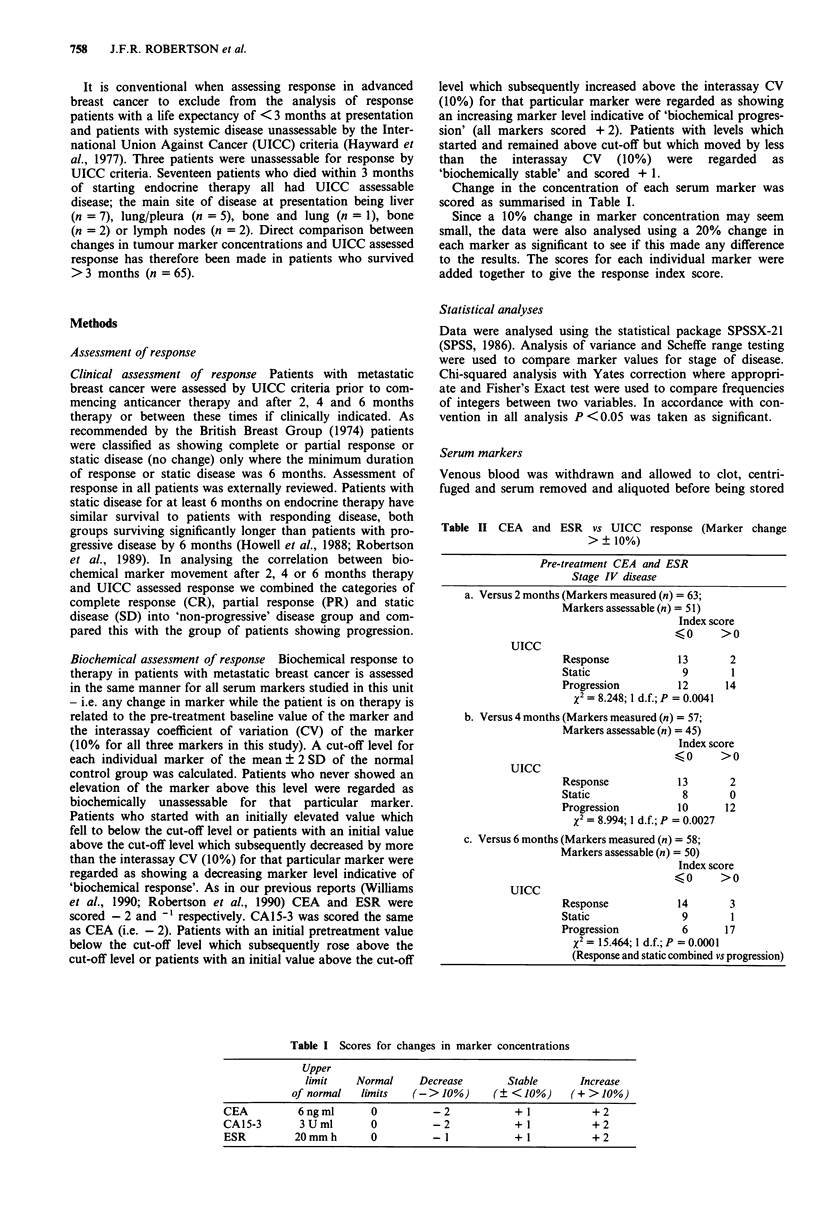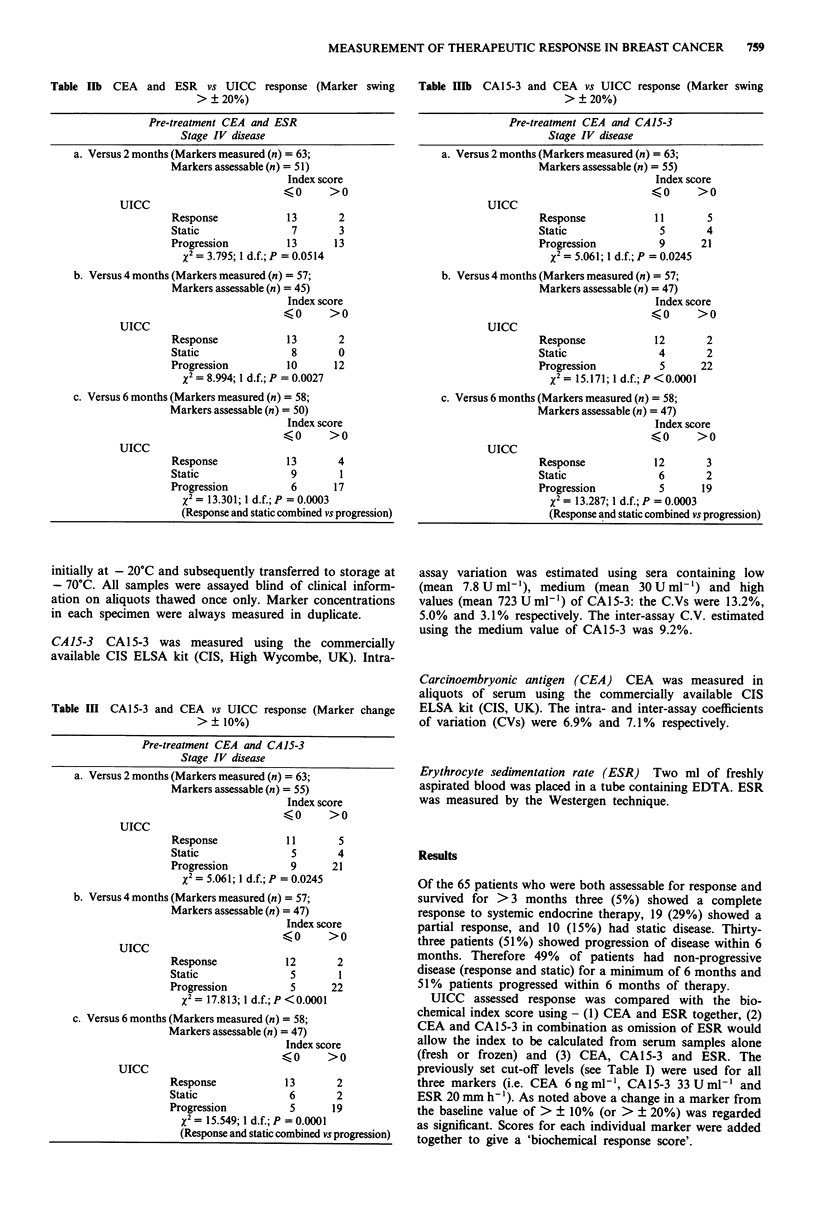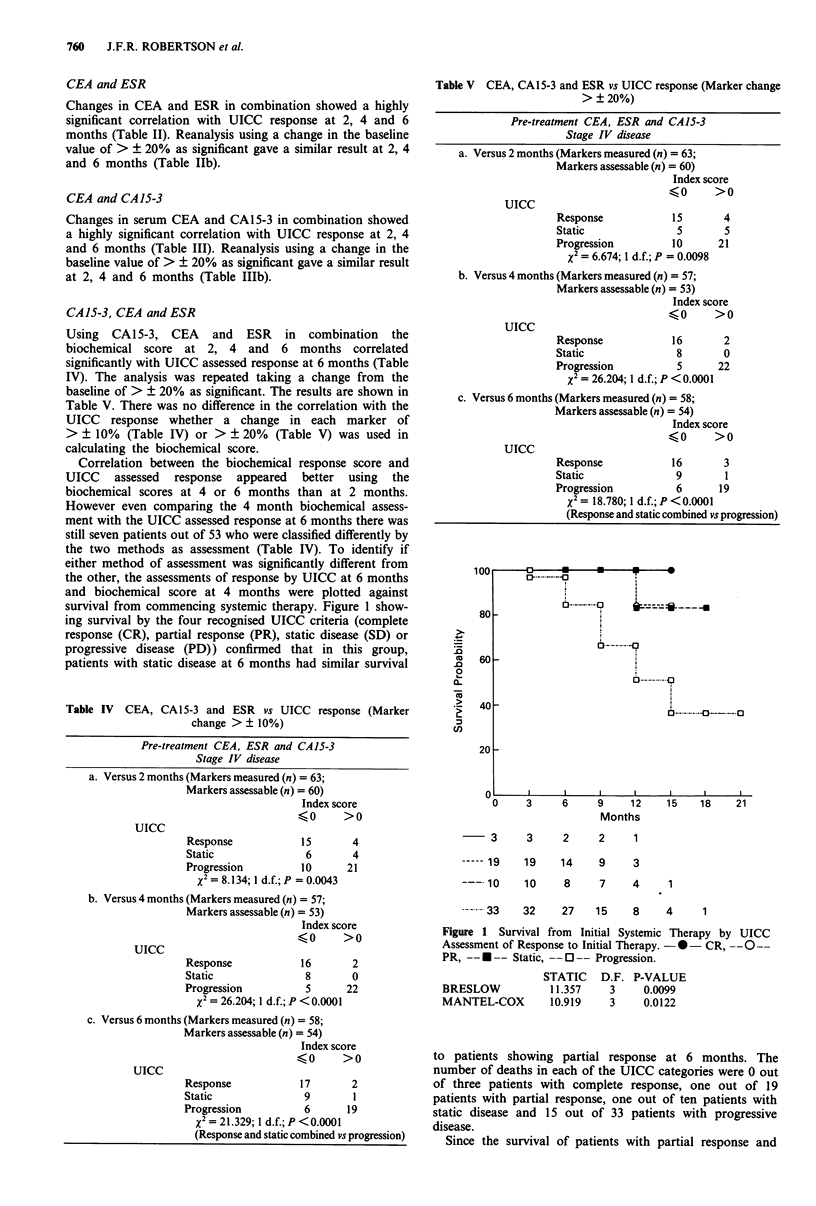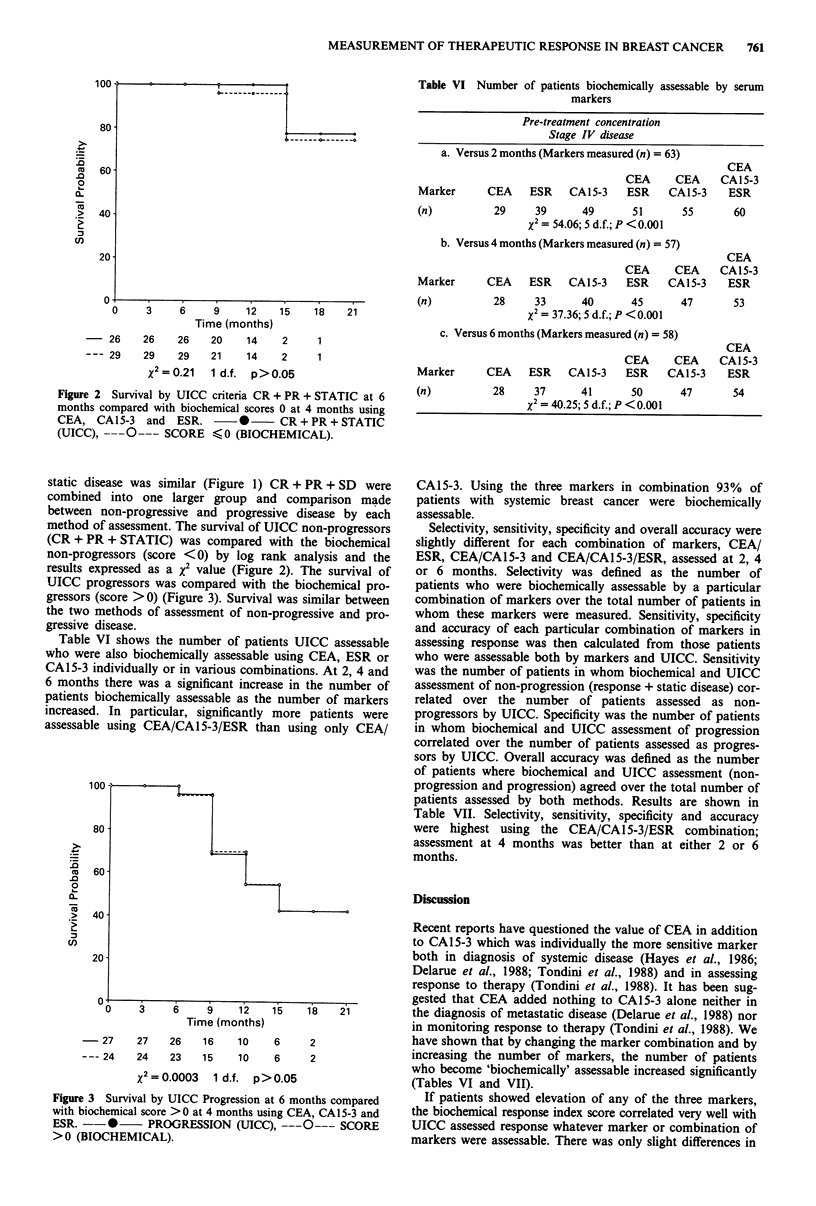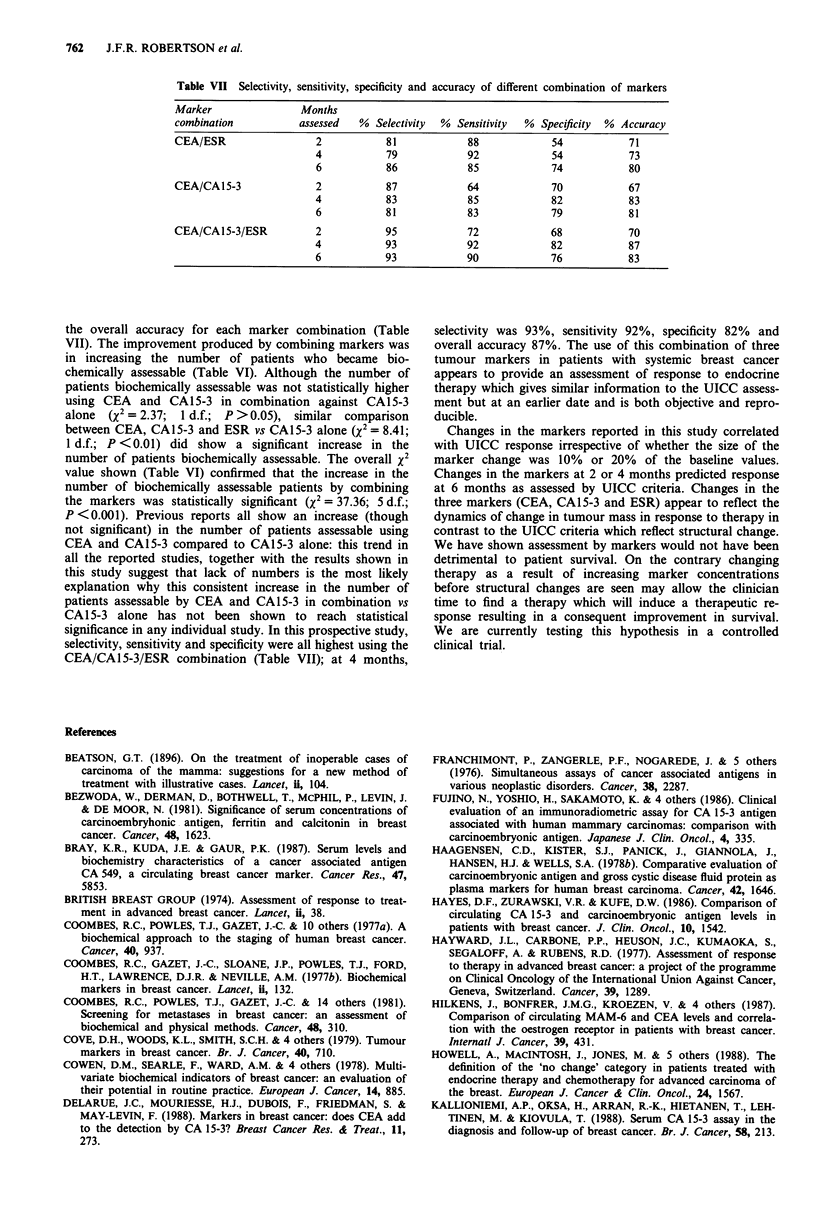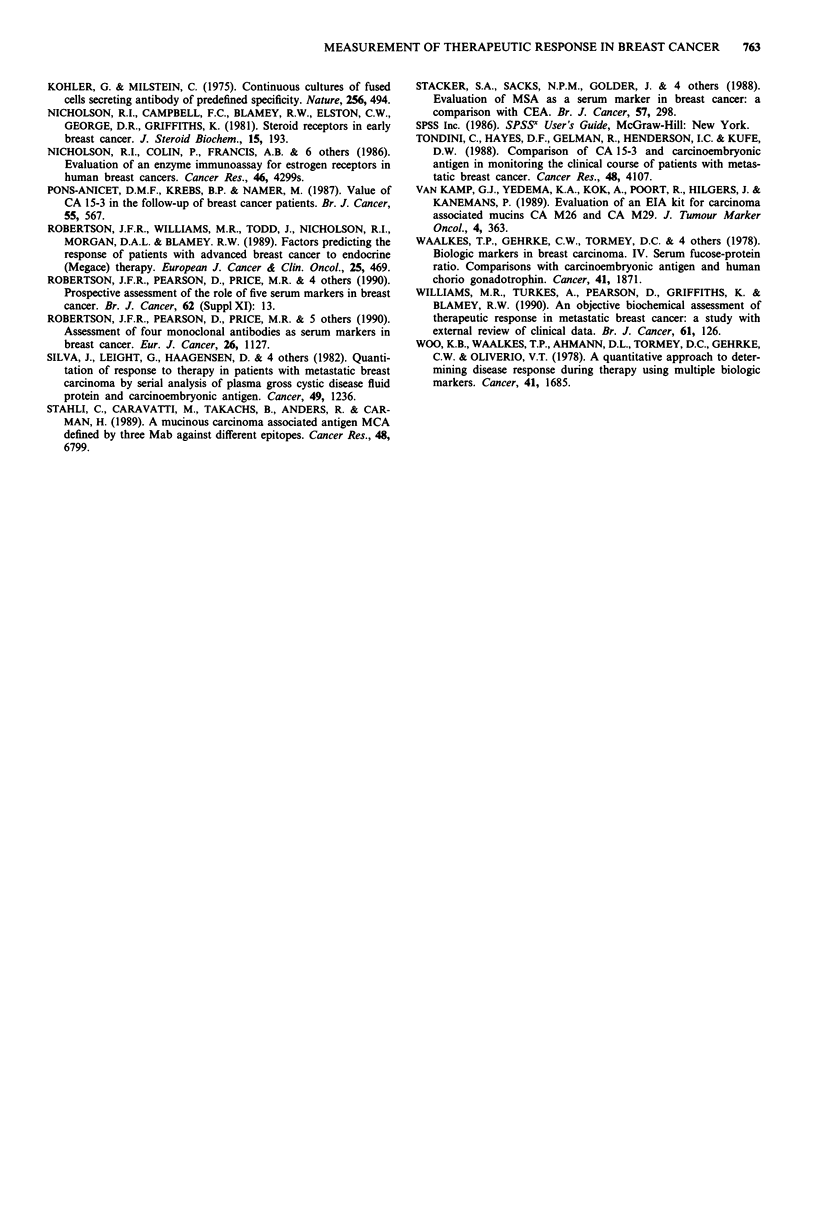# Objective measurement of therapeutic response in breast cancer using tumour markers.

**DOI:** 10.1038/bjc.1991.394

**Published:** 1991-10

**Authors:** J. F. Robertson, D. Pearson, M. R. Price, C. Selby, R. W. Blamey, A. Howell

**Affiliations:** Professional Unit of Surgery, City Hospital, Nottingham, UK.

## Abstract

In 65 patients with systemic breast cancer, a biochemical response index using three tumour markers in combination, carcinoembryonic antigen (CEA), carbohydrate antigen 15-3 (CA 15-3) and erythrocyte sedimentation rate (ESR), allowed objective biochemical assessment of response to endocrine therapy. Changes in these three markers at 2, 4 and 6 months showed a highly significant correlation with UICC assessed response at 6 months. At 4 months, changes in these three markers resulted in a selectivity of 93%, with a sensitivity of 92% and a specificity of 82%. Survival of groups of patients assessed biochemically or by UICC criteria for non-progression or progression showed no significant difference. The advantage of the biochemical assessment are that it is objective and reproducible. The assessment gives similar information to the UICC assessment but can be carried out earlier. Changes in the three markers appears to reflect the dynamics of change in tumour mass in response to systemic therapy in contrast to the UICC criteria which reflect structural change.


Br. J. Cancer (1991), 64, 757-763 © Macmillan Press Ltd., 1991

### Objective measurement of therapeutic response in breast cancer using tumour markers

J.F.R. Robertson[1], D. Pearson[2], M.R. Price[4], C. Selby[3], R.W. Blamey[1] & A. Howell[3]

[1]*Professional Unit of Surgery, City Hospital, Nottingham; Departments of* [2]*Medical Physics and* [3]*Clinical Chemistry, City Hospital;* [4]*Cancer Research Campaign Laboratories, Nottingham;* [5]*Department of Medical Oncology, Christie Hospital, Manchester, UK.*

**Summary** In 65 patients with systemic breast cancer, a biochemical response index using three tumour markers in combination, carcinoembryonic antigen (CEA), carbohydrate antigen 15-3 (CA15-3) and erthrocyte sedimentation rate (ESR), allowed objective biochemical assessment of response to endocrine therapy. Changes in these three markers at 2, 4 and 6 months showed a highly significant correlation with UICC assessed response at 6 months. At 4 months, changes in these three markers resulted in a selectivity of 93%, with a sensitivity of 92% and a specificity of 82%. Survival of groups of patients assessed biochemically or by UICC criteria for non-progression or progression showed no significant difference.

The advantages of the biochemical assessment are that it is objective and reproducible. The assessment gives similar information to the UICC assessment but can be carried out earlier. Changes in the three markers appears to reflect the dynamics of change in tumour mass in response to systemic therapy in contrast to the UICC criteria which reflect structural change.

Endocrine therapy for advanced breast cancer was first introduced by a surgeon in Scotland in 1896 (Beatson, 1896) yet we still lack truly objective criteria by which to assess therapeutic response. Even the criteria most widely used at present, i.e. International Union Against Cancer (UICC) criteria, acknowledge their inherent subjectivity by requiring external review of response data. More recently clinicians have investigated the potential role of tumour markers both in the diagnosis of breast cancer and in measuring response to therapy.

There is no single, ideal tumour marker for breast cancer. Combinations of tumour markers, including carcinoembryonic antigen (CEA), have been investigated to increase the sensitivity of detecting metastases by biological markers (Franchimont *et al.*, 1976; Coombes *et al.*, 1977*a*; Coombes *et al.*, 1977*b*; Cowen *et al.*, 1978; Cove *et al.*, 1979; Coombes *et al.*, 1981; Bezwoda *et al.*, 1981). A much smaller number of studies have reported on the use of CEA in combination with other biological markers in measuring response (Woo *et al.*, 1978; Waalkes *et al.*, 1978; Haagensen *et al.*, 1978; Silva *et al.*, 1982).

Following the discovery of monoclonal antibodies by Kohler and Milstein (1975), monoclonal antibodies have been raised to a number of breast cancer associated antigens. Serum CEA has been examined in combination with a number of monoclonal antibodies raised to tumour associated antigens – e.g. MAM-6 antigen, 115D8 (Hilkens *et al.*, 1987), CA15-3 antigen (Hayes *et al.*, 1986; Fujino *et al.*, 1986; Pons-Anicet *et al.*, 1987; Kallioniemi *et al.*, 1988; Tondini *et al.*, 1988; Delarue *et al.*, 1988) and mammary serum antigen (MSA) (Stacker *et al.*, 1988). Other newer monoclonal antibodies have been reported such as MCA (Stahli *et al.*, 1989), CA 549 (Bray *et al.*, 1989) and CA M26 and CA M29 (van Ramp *et al.*, 1989). However to date the value of these newer monoclonal antibodies either in combination with or compared to CEA has not been reported.

In Nottingham we have previously shown in a retrospective analysis (Williams *et al.*, 1990) and prospectively confirmed (Robertson *et al.*, 1990), that changes in serum CEA and ESR (erythrocyte sedimentation rate) individually correlate with therapeutic response in patients with metastatic breast cancer. We have also evaluated four new serum tumour markers identified by monoclonal antibodies raised either to milk fat globule membrane fractions (HMFG1 and HMFG2) or human breast cancer tissue (CA15-3 and NCRC-11); only changes in serum CA15-3 correlated with therapeutic response (Robertson *et al.*, 1990).

This present study combined the three serum markers showing independent correlation with response (CEA, CA15-3 and ESR) into one index and compared the results with UICC assessed response.

### Patients

Over a 12 month period 85 consecutive patients with newly diagnosed systemic breast cancer presented to our unit. Sixteen patients presented for the first time with stage IV disease while the remaining 69 patients had recurrent metastatic disease having previously been treated for primary breast cancer (Stage I ($n$ = 14), Stage II ($n$ = 44), Stage III ($n$ = 9) and Stage unknown ($n$ = 2)). The mean age (± SD) (years) of the patients was 60.52 (± 12.22) years. Fourteen patients were premenopausal and 71 postmenopausal at the time of diagnosis of metastatic disease. Patients were assessed before treatment and at two monthly intervals by clinical examination, routine haematology and biochemistry, biological markers and skeletal survey. Other investigations (e.g. CT scan) were performed if clinically indicated. The site of initial metastatic disease in these 85 patients was bone ($n$ = 33), lung ($n$ = 27), bone and lung ($n$ = 11) and viscera ($n$ = 14). Oestrogen receptor (ER) and progesterone receptor (PgR) concentration was measured on the primary tumour tissue using either the dextran charcoal method or enzyme immunoassays; both methods have previously been reported by our unit (Nicholson *et al.*, 1981; Nicholson *et al.*, 1986). With both assays values of $\leqslant 5$ fmoles $mg^{-1}$ cytosol protein were regarded as negative. Thirty tumours were ER positive and 23 ER negative. In 32 patients the ER status of the primary tumour was unknown. For PgR 22 tumours were positive, 29 were negative and in 34 PgR status was not known.

All patients received systemic endocrine therapy. Initial therapy for 14 premenopausal patients was the LHRH agonist goserelin, Zoladex (ICI Pharmaceuticals), 3.6 mg monthly by subcutaneous injection and the anti-oestrogen tamoxifen, Nolvadex (ICI Pharmaceuticals), 20 mg b.d. Postmenopausal patients were treated either with tamoxifen 20 mg b.d. ($n$ = 69) or the synthetic progestagen megestrol acetate (Megace, Bristol Myers) 160 mg b.d. ($n$ = 2).

Correspondence: J.F.R. Robertson, Professional Unit of Surgery, City Hospital, Nottingham NG5 1PB, UK.
Received 21 November 1990; and in revised form 26 April 1991.

It is conventional when assessing response in advanced breast cancer to exclude from the analysis of response patients with a life expectancy of $<3$ months at presentation and patients with systemic disease unassessable by the International Union Against Cancer (UICC) criteria (Hayward *et al.*, 1977). Three patients were unassessable for response by UICC criteria. Seventeen patients who died within 3 months of starting endocrine therapy all had UICC assessable disease; the main site of disease at presentation being liver ($n = 7$), lung/pleura ($n = 5$), bone and lung ($n = 1$), bone ($n = 2$) or lymph nodes ($n = 2$). Direct comparison between changes in tumour marker concentrations and UICC assessed response has therefore been made in patients who survived $>3$ months ($n = 65$).

### Methods

### *Assessment of response*

*Clinical assessment of response* Patients with metastatic breast cancer were assessed by UICC criteria prior to commencing anticancer therapy and after 2, 4 and 6 months therapy or between these times if clinically indicated. As recommended by the British Breast Group (1974) patients were classified as showing complete or partial response or static disease (no change) only where the minimum duration of response or static disease was 6 months. Assessment of response in all patients was externally reviewed. Patients with static disease for at least 6 months on endocrine therapy have similar survival to patients with responding disease, both groups surviving significantly longer than patients with progressive disease by 6 months (Howell *et al.*, 1988; Robertson *et al.*, 1989). In analysing the correlation between biochemical marker movement after 2, 4 or 6 months therapy and UICC assessed response we combined the categories of complete response (CR), partial response (PR) and static disease (SD) into 'non-progressive' disease group and compared this with the group of patients showing progression.

*Biochemical assessment of response* Biochemical response to therapy in patients with metastatic breast cancer is assessed in the same manner for all serum markers studied in this unit – i.e. any change in marker while the patient is on therapy is related to the pre-treatment baseline value of the marker and the interassay coefficient of variation (CV) of the marker (10% for all three markers in this study). A cut-off level for each individual marker of the mean $\pm$ 2 SD of the normal control group was calculated. Patients who never showed an elevation of the marker above this level were regarded as biochemically unassessable for that particular marker. Patients who started with an initially elevated value which fell to below the cut-off level or patients with an initial value above the cut-off level which subsequently decreased by more than the interassay CV (10%) for that particular marker were regarded as showing a decreasing marker level indicative of 'biochemical response'. As in our previous reports (Williams *et al.*, 1990; Robertson *et al.*, 1990) CEA and ESR were scored $-2$ and $^{-1}$ respectively. CA15-3 was scored the same as CEA (i.e. $-2$). Patients with an initial pretreatment value below the cut-off level which subsequently rose above the cut-off level or patients with an initial value above the cut-off level which subsequently increased above the interassay CV (10%) for that particular marker were regarded as showing an increasing marker level indicative of 'biochemical progression' (all markers scored $+2$). Patients with levels which started and remained above cut-off but which moved by less than the interassay CV (10%) were regarded as 'biochemically stable' and scored $+1$.

Change in the concentration of each serum marker was scored as summarised in Table I.

Since a 10% change in marker concentration may seem small, the data were also analysed using a 20% change in each marker as significant to see if this made any difference to the results. The scores for each individual marker were added together to give the response index score.

### *Statistical analyses*

Data were analysed using the statistical package SPSSX-21 (SPSS, 1986). Analysis of variance and Scheffe range testing were used to compare marker values for stage of disease. Chi-squared analysis with Yates correction where appropriate and Fisher's Exact test were used to compare frequencies of integers between two variables. In accordance with convention in all analysis $P<0.05$ was taken as significant.

### *Serum markers*

Venous blood was withdrawn and allowed to clot, centrifuged and serum removed and aliquoted before being stored

**Table II** CEA and ESR *vs* UICC response (Marker change $> \pm$ 10%)

| *Pre-treatment CEA and ESR Stage IV disease* | | | |
|---|---|---|---|
| a. Versus 2 months (Markers measured ($n$) = 63; Markers assessable ($n$) = 51) | | | |
| | | Index score $\leqslant 0$ | Index score $>0$ |
| UICC | | | |
| | Response | 13 | 2 |
| | Static | 9 | 1 |
| | Progression | 12 | 14 |
| $\chi^2 = 8.248$; 1 d.f.; $P = 0.0041$ | | | |
| b. Versus 4 months (Markers measured ($n$) = 57; Markers assessable ($n$) = 45) | | | |
| | | Index score $\leqslant 0$ | Index score $>0$ |
| UICC | | | |
| | Response | 13 | 2 |
| | Static | 8 | 0 |
| | Progression | 10 | 12 |
| $\chi^2 = 8.994$; 1 d.f.; $P = 0.0027$ | | | |
| c. Versus 6 months (Markers measured ($n$) = 58; Markers assessable ($n$) = 50) | | | |
| | | Index score $\leqslant 0$ | Index score $>0$ |
| UICC | | | |
| | Response | 14 | 3 |
| | Static | 9 | 1 |
| | Progression | 6 | 17 |
| $\chi^2 = 15.464$; 1 d.f.; $P = 0.0001$ (Response and static combined *vs* progression) | | | |

**Table I** Scores for changes in marker concentrations

| | *Upper limit of normal* | *Normal limits* | *Decrease ($- >10\%$)* | *Stable ($\pm <10\%$)* | *Increase ($+ >10\%$)* |
|---|---|---|---|---|---|
| CEA | 6 ng ml | 0 | $-2$ | $+1$ | $+2$ |
| CA15-3 | 3 U ml | 0 | $-2$ | $+1$ | $+2$ |
| ESR | 20 mm h | 0 | $-1$ | $+1$ | $+2$ |

**Table IIb** CEA and ESR *vs* UICC response (Marker swing $> \pm 20\%$)

| *Pre-treatment CEA and ESR Stage IV disease* | | |
|---|---|---|
| a. Versus 2 months (Markers measured ($n$) = 63; Markers assessable ($n$) = 51) | | |
| | Index score | |
| UICC | $\leqslant 0$ | $>0$ |
| Response | 13 | 2 |
| Static | 7 | 3 |
| Progression | 13 | 13 |
| $\chi^2 = 3.795$; 1 d.f.; $P = 0.0514$ | | |
| b. Versus 4 months (Markers measured ($n$) = 57; Markers assessable ($n$) = 45) | | |
| | Index score | |
| UICC | $\leqslant 0$ | $>0$ |
| Response | 13 | 2 |
| Static | 8 | 0 |
| Progression | 10 | 12 |
| $\chi^2 = 8.994$; 1 d.f.; $P = 0.0027$ | | |
| c. Versus 6 months (Markers measured ($n$) = 58; Markers assessable ($n$) = 50) | | |
| | Index score | |
| UICC | $\leqslant 0$ | $>0$ |
| Response | 13 | 4 |
| Static | 9 | 1 |
| Progression | 6 | 17 |
| $\chi^2 = 13.301$; 1 d.f.; $P = 0.0003$ | | |
| (Response and static combined *vs* progression) | | |

**Table IIIb** CA15-3 and CEA *vs* UICC response (Marker swing $> \pm 20\%$)

| *Pre-treatment CEA and CA15-3 Stage IV disease* | | |
|---|---|---|
| a. Versus 2 months (Markers measured ($n$) = 63; Markers assessable ($n$) = 55) | | |
| | Index score | |
| UICC | $\leqslant 0$ | $>0$ |
| Response | 11 | 5 |
| Static | 5 | 4 |
| Progression | 9 | 21 |
| $\chi^2 = 5.061$; 1 d.f.; $P = 0.0245$ | | |
| b. Versus 4 months (Markers measured ($n$) = 57; Markers assessable ($n$) = 47) | | |
| | Index score | |
| UICC | $\leqslant 0$ | $>0$ |
| Response | 12 | 2 |
| Static | 4 | 2 |
| Progression | 5 | 22 |
| $\chi^2 = 15.171$; 1 d.f.; $P < 0.0001$ | | |
| c. Versus 6 months (Markers measured ($n$) = 58; Markers assessable ($n$) = 47) | | |
| | Index score | |
| UICC | $\leqslant 0$ | $>0$ |
| Response | 12 | 3 |
| Static | 6 | 2 |
| Progression | 5 | 19 |
| $\chi^2 = 13.287$; 1 d.f.; $P = 0.0003$ | | |
| (Response and static combined *vs* progression) | | |

initially at $-20°C$ and subsequently transferred to storage at $-70°C$. All samples were assayed blind of clinical information on aliquots thawed once only. Marker concentrations in each specimen were always measured in duplicate.

*CA15-3* CA15-3 was measured using the commercially available CIS ELSA kit (CIS, High Wycombe, UK). Intra-assay variation was estimated using sera containing low (mean 7.8 U $ml^{-1}$), medium (mean 30 U $ml^{-1}$) and high values (mean 723 U $ml^{-1}$) of CA15-3: the C.Vs were 13.2%, 5.0% and 3.1% respectively. The inter-assay C.V. estimated using the medium value of CA15-3 was 9.2%.

**Table III** CA15-3 and CEA *vs* UICC response (Marker change $> \pm 10\%$)

| *Pre-treatment CEA and CA15-3 Stage IV disease* | | |
|---|---|---|
| a. Versus 2 months (Markers measured ($n$) = 63; Markers assessable ($n$) = 55) | | |
| | Index score | |
| UICC | $\leqslant 0$ | $>0$ |
| Response | 11 | 5 |
| Static | 5 | 4 |
| Progression | 9 | 21 |
| $\chi^2 = 5.061$; 1 d.f.; $P = 0.0245$ | | |
| b. Versus 4 months (Markers measured ($n$) = 57; Markers assessable ($n$) = 47) | | |
| | Index score | |
| UICC | $\leqslant 0$ | $>0$ |
| Response | 12 | 2 |
| Static | 5 | 1 |
| Progression | 5 | 22 |
| $\chi^2 = 17.813$; 1 d.f.; $P < 0.0001$ | | |
| c. Versus 6 months (Markers measured ($n$) = 58; Markers assessable ($n$) = 47) | | |
| | Index score | |
| UICC | $\leqslant 0$ | $>0$ |
| Response | 13 | 2 |
| Static | 6 | 2 |
| Progression | 5 | 19 |
| $\chi^2 = 15.549$; 1 d.f.; $P = 0.0001$ | | |
| (Response and static combined *vs* progression) | | |

*Carcinoembryonic antigen (CEA)* CEA was measured in aliquots of serum using the commercially available CIS ELSA kit (CIS, UK). The intra- and inter-assay coefficients of variation (CVs) were 6.9% and 7.1% respectively.

*Erythrocyte sedimentation rate (ESR)* Two ml of freshly aspirated blood was placed in a tube containing EDTA. ESR was measured by the Westergen technique.

### Results

Of the 65 patients who were both assessable for response and survived for $>3$ months three (5%) showed a complete response to systemic endocrine therapy, 19 (29%) showed a partial response, and 10 (15%) had static disease. Thirty-three patients (51%) showed progression of disease within 6 months. Therefore 49% of patients had non-progressive disease (response and static) for a minimum of 6 months and 51% patients progressed within 6 months of therapy.

UICC assessed response was compared with the biochemical index score using – (1) CEA and ESR together, (2) CEA and CA15-3 in combination as omission of ESR would allow the index to be calculated from serum samples alone (fresh or frozen) and (3) CEA, CA15-3 and ESR. The previously set cut-off levels (see Table I) were used for all three markers (i.e. CEA 6 ng $ml^{-1}$, CA15-3 33 U $ml^{-1}$ and ESR 20 mm $h^{-1}$). As noted above a change in a marker from the baseline value of $> \pm 10\%$ (or $> \pm 20\%$) was regarded as significant. Scores for each individual marker were added together to give a 'biochemical response score'.

### *CEA and ESR*

Changes in CEA and ESR in combination showed a highly significant correlation with UICC response at 2, 4 and 6 months (Table II). Reanalysis using a change in the baseline value of > ± 20% as significant gave a similar result at 2, 4 and 6 months (Table IIb).

### *CEA and CA15-3*

Changes in serum CEA and CA15-3 in combination showed a highly significant correlation with UICC response at 2, 4 and 6 months (Table III). Reanalysis using a change in the baseline value of > ± 20% as significant gave a similar result at 2, 4 and 6 months (Table IIIb).

### *CA15-3, CEA and ESR*

Using CA15-3, CEA and ESR in combination the biochemical score at 2, 4 and 6 months correlated significantly with UICC assessed response at 6 months (Table IV). The analysis was repeated taking a change from the baseline of > ± 20% as significant. The results are shown in Table V. There was no difference in the correlation with the UICC response whether a change in each marker of > ± 10% (Table IV) or > ± 20% (Table V) was used in calculating the biochemical score.

Correlation between the biochemical response score and UICC assessed response appeared better using the biochemical scores at 4 or 6 months than at 2 months. However even comparing the 4 month biochemical assessment with the UICC assessed response at 6 months there was still seven patients out of 53 who were classified differently by the two methods as assessment (Table IV). To identify if either method of assessment was significantly different from the other, the assessments of response by UICC at 6 months and biochemical score at 4 months were plotted against survival from commencing systemic therapy. Figure 1 showing survival by the four recognised UICC criteria (complete response (CR), partial response (PR), static disease (SD) or progressive disease (PD)) confirmed that in this group, patients with static disease at 6 months had similar survival to patients showing partial response at 6 months. The number of deaths in each of the UICC categories were 0 out of three patients with complete response, one out of 19 patients with partial response, one out of ten patients with static disease and 15 out of 33 patients with progressive disease.

Since the survival of patients with partial response and

**Table IV** CEA, CA15-3 and ESR *vs* UICC response (Marker change > ± 10%)

*Pre-treatment CEA, ESR and CA15-3 Stage IV disease*

a. Versus 2 months (Markers measured ($n$) = 63; Markers assessable ($n$) = 60)

| UICC | Index score ≤0 | Index score >0 |
|---|---|---|
| Response | 15 | 4 |
| Static | 6 | 4 |
| Progression | 10 | 21 |

$\chi^2 = 8.134$; 1 d.f.; $P = 0.0043$

b. Versus 4 months (Markers measured ($n$) = 57; Markers assessable ($n$) = 53)

| UICC | Index score ≤0 | Index score >0 |
|---|---|---|
| Response | 16 | 2 |
| Static | 8 | 0 |
| Progression | 5 | 22 |

$\chi^2 = 26.204$; 1 d.f.; $P < 0.0001$

c. Versus 6 months (Markers measured ($n$) = 58; Markers assessable ($n$) = 54)

| UICC | Index score ≤0 | Index score >0 |
|---|---|---|
| Response | 17 | 2 |
| Static | 9 | 1 |
| Progression | 6 | 19 |

$\chi^2 = 21.329$; 1 d.f.; $P < 0.0001$
(Response and static combined *vs* progression)

**Table V** CEA, CA15-3 and ESR *vs* UICC response (Marker change > ± 20%)

*Pre-treatment CEA, ESR and CA15-3 Stage IV disease*

a. Versus 2 months (Markers measured ($n$) = 63; Markers assessable ($n$) = 60)

| UICC | Index score ≤0 | Index score >0 |
|---|---|---|
| Response | 15 | 4 |
| Static | 5 | 5 |
| Progression | 10 | 21 |

$\chi^2 = 6.674$; 1 d.f.; $P = 0.0098$

b. Versus 4 months (Markers measured ($n$) = 57; Markers assessable ($n$) = 53)

| UICC | Index score ≤0 | Index score >0 |
|---|---|---|
| Response | 16 | 2 |
| Static | 8 | 0 |
| Progression | 5 | 22 |

$\chi^2 = 26.204$; 1 d.f.; $P < 0.0001$

c. Versus 6 months (Markers measured ($n$) = 58; Markers assessable ($n$) = 54)

| UICC | Index score ≤0 | Index score >0 |
|---|---|---|
| Response | 16 | 3 |
| Static | 9 | 1 |
| Progression | 6 | 19 |

$\chi^2 = 18.780$; 1 d.f.; $P < 0.0001$
(Response and static combined *vs* progression)

| | | | | | | | |
|---|---|---|---|---|---|---|---|
| —— 3 | 3 | 2 | 2 | 1 | | | |
| ----- 19 | 19 | 14 | 9 | 3 | | | |
| ----·10 | 10 | 8 | 7 | 4 | 1 | | |
| ········ 33 | 32 | 27 | 15 | 8 | 4 | 1 | |

**Figure 1** Survival from Initial Systemic Therapy by UICC Assessment of Response to Initial Therapy. —●— CR, --O-- PR, --■-- Static, --□-- Progression.

| | STATIC | D.F. | P-VALUE |
|---|---|---|---|
| BRESLOW | 11.357 | 3 | 0.0099 |
| MANTEL-COX | 10.919 | 3 | 0.0122 |

**Figure 2** Survival by UICC criteria CR + PR + STATIC at 6 months compared with biochemical scores 0 at 4 months using CEA, CA15-3 and ESR. ——●—— CR + PR + STATIC (UICC), ---O--- SCORE ≤0 (BIOCHEMICAL).

**Table VI** Number of patients biochemically assessable by serum markers

| *Pre-treatment concentration Stage IV disease* | | | | | | |
|---|---|---|---|---|---|---|
| a. Versus 2 months (Markers measured (*n*) = 63) | | | | | | |
| Marker | CEA | ESR | CA15-3 | CEA ESR | CEA CA15-3 | CEA CA15-3 ESR |
| (*n*) | 29 | 39 | 49 | 51 | 55 | 60 |
| | $\chi^2 = 54.06$; 5 d.f.; $P < 0.001$ | | | | | |
| b. Versus 4 months (Markers measured (*n*) = 57) | | | | | | |
| Marker | CEA | ESR | CA15-3 | CEA ESR | CEA CA15-3 | CEA CA15-3 ESR |
| (*n*) | 28 | 33 | 40 | 45 | 47 | 53 |
| | $\chi^2 = 37.36$; 5 d.f.; $P < 0.001$ | | | | | |
| c. Versus 6 months (Markers measured (*n*) = 58) | | | | | | |
| Marker | CEA | ESR | CA15-3 | CEA ESR | CEA CA15-3 | CEA CA15-3 ESR |
| (*n*) | 28 | 37 | 41 | 50 | 47 | 54 |
| | $\chi^2 = 40.25$; 5 d.f.; $P < 0.001$ | | | | | |

static disease was similar (Figure 1) CR + PR + SD were combined into one larger group and comparison made between non-progressive and progressive disease by each method of assessment. The survival of UICC non-progressors (CR + PR + STATIC) was compared with the biochemical non-progressors (score <0) by log rank analysis and the results expressed as a $\chi^2$ value (Figure 2). The survival of UICC progressors was compared with the biochemical progressors (score >0) (Figure 3). Survival was similar between the two methods of assessment of non-progressive and progressive disease.

Table VI shows the number of patients UICC assessable who were also biochemically assessable using CEA, ESR or CA15-3 individually or in various combinations. At 2, 4 and 6 months there was a significant increase in the number of patients biochemically assessable as the number of markers increased. In particular, significantly more patients were assessable using CEA/CA15-3/ESR than using only CEA/CA15-3. Using the three markers in combination 93% of patients with systemic breast cancer were biochemically assessable.

**Figure 3** Survival by UICC Progression at 6 months compared with biochemical score >0 at 4 months using CEA, CA15-3 and ESR. ——●—— PROGRESSION (UICC), ---O--- SCORE >0 (BIOCHEMICAL).

Selectivity, sensitivity, specificity and overall accuracy were slightly different for each combination of markers, CEA/ESR, CEA/CA15-3 and CEA/CA15-3/ESR, assessed at 2, 4 or 6 months. Selectivity was defined as the number of patients who were biochemically assessable by a particular combination of markers over the total number of patients in whom these markers were measured. Sensitivity, specificity and accuracy of each particular combination of markers in assessing response was then calculated from those patients who were assessable both by markers and UICC. Sensitivity was the number of patients in whom biochemical and UICC assessment of non-progression (response + static disease) correlated over the number of patients assessed as non-progressors by UICC. Specificity was the number of patients in whom biochemical and UICC assessment of progression correlated over the number of patients assessed as progressors by UICC. Overall accuracy was defined as the number of patients where biochemical and UICC assessment (non-progression and progression) agreed over the total number of patients assessed by both methods. Results are shown in Table VII. Selectivity, sensitivity, specificity and accuracy were highest using the CEA/CA15-3/ESR combination; assessment at 4 months was better than at either 2 or 6 months.

### Discussion

Recent reports have questioned the value of CEA in addition to CA15-3 which was individually the more sensitive marker both in diagnosis of systemic disease (Hayes *et al.*, 1986; Delarue *et al.*, 1988; Tondini *et al.*, 1988) and in assessing response to therapy (Tondini *et al.*, 1988). It has been suggested that CEA added nothing to CA15-3 alone neither in the diagnosis of metastatic disease (Delarue *et al.*, 1988) nor in monitoring response to therapy (Tondini *et al.*, 1988). We have shown that by changing the marker combination and by increasing the number of markers, the number of patients who become 'biochemically' assessable increased significantly (Tables VI and VII).

If patients showed elevation of any of the three markers, the biochemical response index score correlated very well with UICC assessed response whatever marker or combination of markers were assessable. There was only slight differences in

Table VII Selectivity, sensitivity, specificity and accuracy of different combination of markers

| Marker combination | Months assessed | % Selectivity | % Sensitivity | % Specificity | % Accuracy |
|---|---|---|---|---|---|
| CEA/ESR | 2 | 81 | 88 | 54 | 71 |
| | 4 | 79 | 92 | 54 | 73 |
| | 6 | 86 | 85 | 74 | 80 |
| CEA/CA15-3 | 2 | 87 | 64 | 70 | 67 |
| | 4 | 83 | 85 | 82 | 83 |
| | 6 | 81 | 83 | 79 | 81 |
| CEA/CA15-3/ESR | 2 | 95 | 72 | 68 | 70 |
| | 4 | 93 | 92 | 82 | 87 |
| | 6 | 93 | 90 | 76 | 83 |

the overall accuracy for each marker combination (Table VII). The improvement produced by combining markers was in increasing the number of patients who became biochemically assessable (Table VI). Although the number of patients biochemically assessable was not statistically higher using CEA and CA15-3 in combination against CA15-3 alone ($\chi^2 = 2.37$; 1 d.f.; $P > 0.05$), similar comparison between CEA, CA15-3 and ESR *vs* CA15-3 alone ($\chi^2 = 8.41$; 1 d.f.; $P < 0.01$) did show a significant increase in the number of patients biochemically assessable. The overall $\chi^2$ value shown (Table VI) confirmed that the increase in the number of biochemically assessable patients by combining the markers was statistically significant ($\chi^2 = 37.36$; 5 d.f.; $P < 0.001$). Previous reports all show an increase (though not significant) in the number of patients assessable using CEA and CA15-3 compared to CA15-3 alone: this trend in all the reported studies, together with the results shown in this study suggest that lack of numbers is the most likely explanation why this consistent increase in the number of patients assessable by CEA and CA15-3 in combination *vs* CA15-3 alone has not been shown to reach statistical significance in any individual study. In this prospective study, selectivity, sensitivity and specificity were all highest using the CEA/CA15-3/ESR combination (Table VII); at 4 months, selectivity was 93%, sensitivity 92%, specificity 82% and overall accuracy 87%. The use of this combination of three tumour markers in patients with systemic breast cancer appears to provide an assessment of response to endocrine therapy which gives similar information to the UICC assessment but at an earlier date and is both objective and reproducible.

Changes in the markers reported in this study correlated with UICC response irrespective of whether the size of the marker change was 10% or 20% of the baseline values. Changes in the markers at 2 or 4 months predicted response at 6 months as assessed by UICC criteria. Changes in the three markers (CEA, CA15-3 and ESR) appear to reflect the dynamics of change in tumour mass in response to therapy in contrast to the UICC criteria which reflect structural change. We have shown assessment by markers would not have been detrimental to patient survival. On the contrary changing therapy as a result of increasing marker concentrations before structural changes are seen may allow the clinician time to find a therapy which will induce a therapeutic response resulting in a consequent improvement in survival. We are currently testing this hypothesis in a controlled clinical trial.

### References

BEATSON, G.T. (1896). On the treatment of inoperable cases of carcinoma of the mamma: suggestions for a new method of treatment with illustrative cases. *Lancet*, **ii**, 104.

BEZWODA, W., DERMAN, D., BOTHWELL, T., MCPHIL, P., LEVIN, J. & DE MOOR, N. (1981). Significance of serum concentrations of carcinoembryhonic antigen, ferritin and calcitonin in breast cancer. *Cancer*, **48**, 1623.

BRAY, K.R., KUDA, J.E. & GAUR, P.K. (1987). Serum levels and biochemistry characteristics of a cancer associated antigen CA 549, a circulating breast cancer marker. *Cancer Res.*, **47**, 5853.

BRITISH BREAST GROUP (1974). Assessment of response to treatment in advanced breast cancer. *Lancet*, **ii**, 38.

COOMBES, R.C., POWLES, T.J., GAZET, J.-C. & 10 others (1977*a*). A biochemical approach to the staging of human breast cancer. *Cancer*, **40**, 937.

COOMBES, R.C., GAZET, J.-C., SLOANE, J.P., POWLES, T.J., FORD, H.T., LAWRENCE, D.J.R. & NEVILLE, A.M. (1977*b*). Biochemical markers in breast cancer. *Lancet*, **ii**, 132.

COOMBES, R.C., POWLES, T.J., GAZET, J.-C. & 14 others (1981). Screening for metastases in breast cancer: an assessment of biochemical and physical methods. *Cancer*, **48**, 310.

COVE, D.H., WOODS, K.L., SMITH, S.C.H. & 4 others (1979). Tumour markers in breast cancer. *Br. J. Cancer*, **40**, 710.

COWEN, D.M., SEARLE, F., WARD, A.M. & 4 others (1978). Multivariate biochemical indicators of breast cancer: an evaluation of their potential in routine practice. *European J. Cancer*, **14**, 885.

DELARUE, J.C., MOURIESSE, H.J., DUBOIS, F., FRIEDMAN, S. & MAY-LEVIN, F. (1988). Markers in breast cancer: does CEA add to the detection by CA 15-3? *Breast Cancer Res. & Treat.*, **11**, 273.

FRANCHIMONT, P., ZANGERLE, P.F., NOGAREDE, J. & 5 others (1976). Simultaneous assays of cancer associated antigens in various neoplastic disorders. *Cancer*, **38**, 2287.

FUJINO, N., YOSHIO, H., SAKAMOTO, K. & 4 others (1986). Clinical evaluation of an immunoradiometric assay for CA 15-3 antigen associated with human mammary carcinomas: comparison with carcinoembryonic antigen. *Japanese J. Clin. Oncol.*, **4**, 335.

HAAGENSEN, C.D., KISTER, S.J., PANICK, J., GIANNOLA, J., HANSEN, H.J. & WELLS, S.A. (1978*b*). Comparative evaluation of carcinoembryonic antigen and gross cystic disease fluid protein as plasma markers for human breast carcinoma. *Cancer*, **42**, 1646.

HAYES, D.F., ZURAWSKI, V.R. & KUFE, D.W. (1986). Comparison of circulating CA 15-3 and carcinoembryonic antigen levels in patients with breast cancer. *J. Clin. Oncol.*, **10**, 1542.

HAYWARD, J.L., CARBONE, P.P., HEUSON, J.C., KUMAOKA, S., SEGALOFF, A. & RUBENS, R.D. (1977). Assessment of response to therapy in advanced breast cancer: a project of the programme on Clinical Oncology of the International Union Against Cancer, Geneva, Switzerland. *Cancer*, **39**, 1289.

HILKENS, J., BONFRER, J.M.G., KROEZEN, V. & 4 others (1987). Comparison of circulating MAM-6 and CEA levels and correlation with the oestrogen receptor in patients with breast cancer. *Internatl J. Cancer*, **39**, 431.

HOWELL, A., MACINTOSH, J., JONES, M. & 5 others (1988). The definition of the 'no change' category in patients treated with endocrine therapy and chemotherapy for advanced carcinoma of the breast. *European J. Cancer & Clin. Oncol.*, **24**, 1567.

KALLIONIEMI, A.P., OKSA, H., ARRAN, R.-K., HIETANEN, T., LEHTINEN, M. & KIOVULA, T. (1988). Serum CA 15-3 assay in the diagnosis and follow-up of breast cancer. *Br. J. Cancer*, **58**, 213.

KOHLER, G. & MILSTEIN, C. (1975). Continuous cultures of fused cells secreting antibody of predefined specificity. *Nature*, **256**, 494.

NICHOLSON, R.I., CAMPBELL, F.C., BLAMEY, R.W., ELSTON, C.W., GEORGE, D.R., GRIFFITHS, K. (1981). Steroid receptors in early breast cancer. *J. Steroid Biochem.*, **15**, 193.

NICHOLSON, R.I., COLIN, P., FRANCIS, A.B. & 6 others (1986). Evaluation of an enzyme immunoassay for estrogen receptors in human breast cancers. *Cancer Res.*, **46**, 4299s.

PONS-ANICET, D.M.F., KREBS, B.P. & NAMER, M. (1987). Value of CA 15-3 in the follow-up of breast cancer patients. *Br. J. Cancer*, **55**, 567.

ROBERTSON, J.F.R., WILLIAMS, M.R., TODD, J., NICHOLSON, R.I., MORGAN, D.A.L. & BLAMEY. R.W. (1989). Factors predicting the response of patients with advanced breast cancer to endocrine (Megace) therapy. *European J. Cancer & Clin. Oncol.*, **25**, 469.

ROBERTSON, J.F.R., PEARSON, D., PRICE, M.R. & 4 others (1990). Prospective assessment of the role of five serum markers in breast cancer. *Br. J. Cancer*, **62** (Suppl XI): 13.

ROBERTSON, J.F.R., PEARSON, D., PRICE, M.R. & 5 others (1990). Assessment of four monoclonal antibodies as serum markers in breast cancer. *Eur. J. Cancer*, **26**, 1127.

SILVA, J., LEIGHT, G., HAAGENSEN, D. & 4 others (1982). Quantitation of response to therapy in patients with metastatic breast carcinoma by serial analysis of plasma gross cystic disease fluid protein and carcinoembryonic antigen. *Cancer*, **49**, 1236.

STAHLI, C., CARAVATTI, M., TAKACHS, B., ANDERS, R. & CARMAN, H. (1989). A mucinous carcinoma associated antigen MCA defined by three Mab against different epitopes. *Cancer Res.*, **48**, 6799.

STACKER, S.A., SACKS, N.P.M., GOLDER, J. & 4 others (1988). Evaluation of MSA as a serum marker in breast cancer: a comparison with CEA. *Br. J. Cancer*, **57**, 298.

SPSS Inc. (1986). *SPSS$^x$ User's Guide*, McGraw-Hill: New York.

TONDINI, C., HAYES, D.F., GELMAN, R., HENDERSON, I.C. & KUFE, D.W. (1988). Comparison of CA 15-3 and carcinoembryonic antigen in monitoring the clinical course of patients with metastatic breast cancer. *Cancer Res.*, **48**, 4107.

VAN KAMP, G.J., YEDEMA, K.A., KOK, A., POORT, R., HILGERS, J. & KANEMANS, P. (1989). Evaluation of an EIA kit for carcinoma associated mucins CA M26 and CA M29. *J. Tumour Marker Oncol.*, **4**, 363.

WAALKES, T.P., GEHRKE, C.W., TORMEY, D.C. & 4 others (1978). Biologic markers in breast carcinoma. IV. Serum fucose-protein ratio. Comparisons with carcinoembryonic antigen and human chorio gonadotrophin. *Cancer*, **41**, 1871.

WILLIAMS, M.R., TURKES, A., PEARSON, D., GRIFFITHS, K. & BLAMEY, R.W. (1990). An objective biochemical assessment of therapeutic response in metastatic breast cancer: a study with external review of clinical data. *Br. J. Cancer*, **61**, 126.

WOO, K.B., WAALKES, T.P., AHMANN, D.L., TORMEY, D.C., GEHRKE, C.W. & OLIVERIO, V.T. (1978). A quantitative approach to determining disease response during therapy using multiple biologic markers. *Cancer*, **41**, 1685.